# GWO and WOA variable step MPPT algorithms-based PV system output power optimization

**DOI:** 10.1038/s41598-025-89898-x

**Published:** 2025-03-06

**Authors:** Abderrahim Zemmit, Abdelouadoud Loukriz, Khaled Belhouchet, Yahya Z. Alharthi, Muhannad Alshareef, Prabhu Paramasivam, Sherif S. M. Ghoneim

**Affiliations:** 1 Electrical Engineering Department, Electrical Engineering Laboratory (LGE), University of M’Sila, M’Sila, 28000 Algeria; 2 Electrical Engineering Department, University of Science and Technology Houari Boumediene, Alger, 1611 Algeria; 3 Department of Electrical Engineering, College of Engineering, University of Hafr Albatin, Hafr Al Batin, 39524 Saudi Arabia; 4 Department of Electrical Engineering, College of Engineering and Computing in Al-Qunfudhah, Umm al-Qura University, Mecca, Saudi Arabia; 5 Department of Research and Innovation, Saveetha School of Engineering, SIMATS, Chennai, 602105 India; 6 Department of Mechanical Engineering, Mattu University, Mettu, 318 Ethiopia; 7 Department of Electrical Engineering, College of Engineering, Taif University, Taif, 21944 Saudi Arabia

**Keywords:** Variable step size MPPT algorithm, MPPT, Optimization, GWO, WOA, Engineering, Electrical and electronic engineering

## Abstract

The nonlinear characteristics and low efficiency of photovoltaic (PV) systems remain critical challenges that necessitate advanced solutions. This study proposes two innovative Maximum Power Point Tracking (MPPT) algorithms based on the Whale Optimization Algorithm (WOA) and Grey Wolf Optimization (GWO). The primary advantage of these methods lies in their adaptive step-size optimization, leveraging multiple criteria to determine the optimal step size. A novel fitness function was developed to improve tracking accuracy, minimize ripple, and reduce overshoot. Simulation results demonstrated remarkable improvements, including up to 98% reduction in ripple, 67% reduction in overshoot, and significant improvements in tracking accuracy compared to fixed-step methods. Field validation was conducted using real-world data from the Ain El Melh PV station in Algeria on June 21, 2023. Experimental results confirmed the effectiveness of the proposed methods, with the WOA-based MPPT achieving up to 99% ripple reduction and 40% overshoot reduction under dynamic environmental conditions. A comparative analysis of MPPT algorithms revealed superior performance metrics for the bio-inspired methods. The PO-WOA algorithm achieved the highest efficiency of 98.87% in simulation and 98.94% in real data, surpassing both PO and PO-GWO. It also minimized power loss to 0.56 W in simulation and 0.39 W in real data, demonstrating its optimization capabilities under fluctuating conditions. Although its response time was slightly longer than other methods, at 0.65 s in simulation and 0.48 s in real data, it prioritized stability and precision. These findings underscore the potential of WOA and GWO algorithms to enhance PV system performance, offering robust and efficient solutions for optimizing energy output in both simulation and real-world scenarios.

## Introduction

Recent surges in global energy consumption have significantly exceeded previous forecasts, driven by rapid industrialization, urbanization, and population growth^1–3^. Increased demand has increased pressure on existing energy resources, escalating prices across the energy sector. In light of these developments, there has been a compelling shift in focus towards renewable energy sources as viable and sustainable alternatives to conventional fossil fuels^4^. The urgency of transitioning to renewables has been underscored by the growing recognition of climate change and the pressing need to reduce greenhouse gas emissions associated with fossil fuel use^5^. Renewable energy sources encompass solar, wind, hydro, and geothermal power., present the potential for abundant supply, and offer compelling advantages over traditional energy systems^6^. These advantages include significantly reducing environmental pollution, as renewables emit little to no greenhouse gases during operation, thus contributing to cleaner air and a healthier ecosystem. Furthermore, Renewable energy systems can bolster energy security by broadening the range of energy sources lessening reliance on imported fuels., and stabilizing energy prices in the long term^7,8^.

Among the various renewable energy options available, photovoltaic (PV) energy stands out for its simplicity and user-friendly characteristics. PV systems are relatively easy to install and maintain, making them accessible for both residential and commercial applications^9^. Their low maintenance requirements, combined with the decreasing costs of solar panels and related technologies, have made solar energy an increasingly attractive investment. Moreover, PV systems can provide reliable and consistent performance, even in decentralized settings, which is particularly beneficial in remote or rural areas lacking access to traditional energy grids^10^.

PV energy is valued for its simplicity, low upkeep, cost-efficiency, and strong performance. However, despite its potential as a renewable energy source, it faces challenges such as low conversion efficiency (9–17%) and non-linear behavior^11^.

Consequently, numerous studies and research endeavors on developing and enhancing PV systems have continuously refined, particularly in fields encompassing efficiency, MPPT methods, DC/DC converters, cell materials, and other relevant topics.

Maximum power tracking (MPPT) algorithms have been extensively investigated^12,13^ to enhance the efficiency of PV systems. These algorithms can be categorized into either “traditional” or “intelligent” techniques. Traditional methods include “perturb and observe” (P&O), “incremental conductance” (IC)^14^, “hill climbing” (HC), “fractional open-circuit voltage” (FOCV), and “fractional short-circuit current” (FSCI)^15^. In contrast, smart methods utilize approaches such as neural networks, Grey Wolf Optimization (GWO), fuzzy logic^16^, PSO, and GA^17^.

Traditional MPPT algorithms often struggle to distinguish Global Maximum Power Point (GMPP) on the P-V curve from other Local Maximum Power Points (LMPPs) due to the wide range of LMPPs. Sophisticated MPPT algorithms have been proposed to overcome this challenge and extract maximum efficiency from PV systems^18^. Researchers have proposed various strategies and methodologies in the literature to address these limitations, particularly for Perturb and Seek Control (PSC). One such technique, introduced by^19^, employs a two-step approach based on the GMPP tracking algorithm, demonstrating superior tracking performance compared to the particle swarm optimization (PSO) algorithm in PSCs. In^20^, The authors introduced a prediction model based on a natural cubic spline, and their algorithm for predicting the MPP is now a standard component of the iterative search. The temperature-based MPPT sensor introduced by^21^ is a novel, high-tech addition to the field. This technique takes advantage of the notion that voltage output by a module concerning to the temperature at the surface on a PV board. In^17^Yang et al. outlined a comprehensive overview of at least 40 distinct approaches, encompassing a range of sophisticated classical methods such as the three-point weight comparison method, the parasitic comparison method, and intelligent and optimized procedures. However, this work focuses exclusively on comparing those five factors as they pertain to tracking algorithms. Based on the type of tracking, Mukherjee et al.^22^drew a comparative study. Mathematical calculations/metaheuristics MPPT comparisons. The right MPPT becomes important in the course of the entire design process. Several MPP factors have been separated in the literature^20^.

Dong Mi and Thamer significantly advanced the PV system by developing a composite MPPT control algorithm. This algorithm ingeniously integrates the well-established method of incremental conductance with an improved variant of the Particle Swarm Optimization (PSO) algorithm^23,24^. The composite nature of this approach leverages the strengths of both methods: incremental conductance provides a robust mechanism for tracking the maximum power point under steady conditions. At the same time, the PSO algorithm enhances the system’s ability to adapt to more dynamic and non-linear scenarios. By synergizing these two methods, the researchers significantly improved the MPPT system’s tracking accuracy, ensuring that the PV array operates consistently at its optimal power output.

However, despite the improvements in accuracy, the algorithm is not without its challenges. One of the primary drawbacks arises when the environmental conditions, such as solar irradiance and temperature, change abruptly. Under such circumstances, the PSO component of the algorithm is required to initiate a global search across the entire solution space to locate the new maximum power point. This global search, while effective, is computationally intensive and places a substantial load on the system’s processing capabilities. Consequently, this leads to a reduction in the response speed of the system, as the computational resources are heavily taxed. This trade-off between accuracy and speed remains critical for further research and optimization. In 2018, Chunjuan Liu offered a novel algorithm for MPPT control designed to address multi-peak power tracking challenges commonly encountered in PV systems. This algorithm is based on the Slime Mould Optimization Algorithm (SMA), a bio-inspired technique that simulates the natural behaviour of slime moulds^25^. Slime moulds are fascinating organisms known for their ability to navigate complex environments in search of food, exhibiting behaviours such as diffusion and foraging. By modelling these behaviours, the SMA-based algorithm can dynamically adjust its search patterns in response to changing environmental conditions. The SMA technique computes the searching space formed by each weight and probabilistic function, enabling the slime mold to traverse the optimization landscape in all directions and with any step size. Such flexibility enables the algorithm to prevent becoming trapped in local optima, a common problem in traditional MPPT methods, and instead continue searching for the global maximum power point. Moreover, the algorithm is particularly efficient at maintaining performance even when a sudden change in light intensity occurs, such as during passing clouds or partial shading. However, the downside of this sophisticated search mechanism is that it requires a large amount of computational power, which can reduce the system’s overall efficiency. The significant data processing demands of the algorithm may lead to delays in real-time operation, thus impacting the responsiveness of the system in case of rapid variations in environmental settings.

In 2020, Zongyang Cui introduced another innovative approach to MPPT for PV systems, which combined the strengths of a hybrid improved Bat Algorithm with a fuzzy logic control system^26^. The Bat Algorithm, inspired by the echolocation behavior of bats, is a population-based metaheuristic that has shown considerable promise in solving complex optimization problems. The hybrid version of this algorithm, as proposed by Cui, incorporates improvements that enhance its ability to explore and exploit the search space effectively. Combined with a fuzzy logic system, the hybrid Bat Algorithm exhibits high adaptability, making it well-suited to the inherently variable nature of solar energy. The fuzzy logic component allows the system to handle imprecise inputs and make decisions based on possible scenarios, rather than relying on fixed thresholds or rigid rules. This adaptability is particularly advantageous when dealing with the fluctuating conditions typical of solar energy systems. However, the implementation of this approach is not without its challenges. The hybrid algorithm requires the cooperative adjustment of multiple factors, including fine-tuning the Bat Algorithm parameters and the fuzzy logic rules. This complexity in execution can make the system difficult to implement, requiring careful calibration and potentially leading to increased development and operational costs.

Furthermore, Sabaripandiyan, D. made a notable contribution to the field by enhancing the traditional incremental conductance method by introducing a variable step conductance increment strategy, coupled with the control of a tracking scale factor^27^. This refined algorithm was specifically designed to address one of the most persistent challenges in MPPT: the trade-off between response speed and steady-state accuracy. By allowing the step size of the conductance increment to vary dynamically, the algorithm can achieve a faster response to changes in environmental conditions without sacrificing the accuracy of the steady-state tracking. However, this approach introduces new complexities, particularly in selecting the step adjustment coefficient, denoted as S(k). Determining an optimal S(k) is critical for balancing the algorithm’s performance. Yet, it involves a complex decision-making process that requires a deep understanding of the system’s behaviour under various conditions. The intricate nature of this selection process suggests that there is still room for further refinement and optimization to maximize the algorithm’s effectiveness across a broader range of operating conditions.

Recent advancements in MPPT techniques for PV systems have focused on adaptive and hybrid control methods to address challenges posed by fluctuating environmental conditions, such as variable solar irradiance and partial shading. For instance, an Enhanced MPPT approach combining P&O with an Enhanced Model Reference Adaptive Controller (EMRAC) has demonstrated 98.28% tracking efficiency and rapid convergence (0.11 s) for grid-integrated PV systems^28^. Similarly, a Lyapunov-Based Model Reference Adaptive Control (LB-MRAC) has achieved 99.15–99.59% efficiency with a tracking speed of 3.7 ms under highly dynamic and stochastic weather conditions^29^. Hybrid two-stage MPPT methods, such as those integrating Modified Model Reference Adaptive Controllers (MMRAC), have shown robust performance under grid-integrated and partial shading scenarios, with significant improvements in tracking accuracy, ripple reduction, and error minimization^30^. Furthermore, the Adjustable Variable Step-Based MRAC MPPT technique has demonstrated exceptional performance in highly fluctuating and cloudy conditions, achieving tracking efficiency between 99.26% and 99.70% with negligible ripples^31^.

While these various MPPT algorithms represent significant advancements in the field of PV energy systems, they also highlight the ongoing challenges and trade-offs involved in optimizing system performance. Each method offers unique advantages, whether in accuracy, adaptability, or speed, but also comes with limitations, particularly in computational demands and implementation complexity. As research in this area continues to evolve, further innovations will likely focus on overcoming these limitations to develop more efficient, responsive, and practical solutions for maximizing the power output of PV systems.

Despite the advantages of the above research, these methods are prone to oscillations around the MPP and lack robustness under rapidly changing weather conditions. In addition, there is a notable gap in research concerning the optimization of step size, which is essential for balancing the trade-off between speed of response and stability in MPPT operations. This study makes two primary contributions to the field of MPPT for PV systems:

- A novel objective function is introduced to optimize MPPT accuracy while simultaneously minimizing overshoot and ripple effects. This approach represents a significant advancement over traditional methods by integrating multiple performance criteria into a unified optimization framework.
- A comprehensive comparative analysis is conducted between the conventional PO fixed step method and the proposed GWO and WOA variable step methods. This comparative study evaluates their effectiveness under various operational conditions, highlighting the superior performance of the GWO and WOA algorithms in optimizing MPPT efficiency under dynamic environmental factors.
- The study also investigates real data validation, providing insights derived from experimental results to further support the effectiveness of the proposed methods.

The remainder of the paper is structured as follows: section “Modeling of photovoltaic cell” provides a discussion on the modeling of a PV cell, introducing the fundamental equations and principles. Section “Conventional (P&O) MPPT” presents the conventional Perturb and Observe (P&O) MPPT technique, outlining its mechanism and implementation. Section “Bio-inspired optimization algorithms: GWO and WOA” introduces bio-inspired optimization algorithms, specifically GWO and WOA, detailing their theoretical foundations and application in MPPT for PV systems. Section “MPPT algorithm with variable step size using GWO and WON” describes the implementation of an MPPT algorithm with a variable step size using both WOA and GWO, including the system setup, objective functions, and optimization strategies. Section “Results and discussion” presents the results and analysis, comparing the performance of the proposed methods with traditional MPPT algorithms. Section Real data investigation investigates real data validation, providing insights from experimental results. Finally, section “Comparative analysis of MPPT algorithm performance metrics” concludes the paper, summarizing key findings and suggesting potential avenues for future research.

## Modeling of photovoltaic cell

**Fig. 1:**
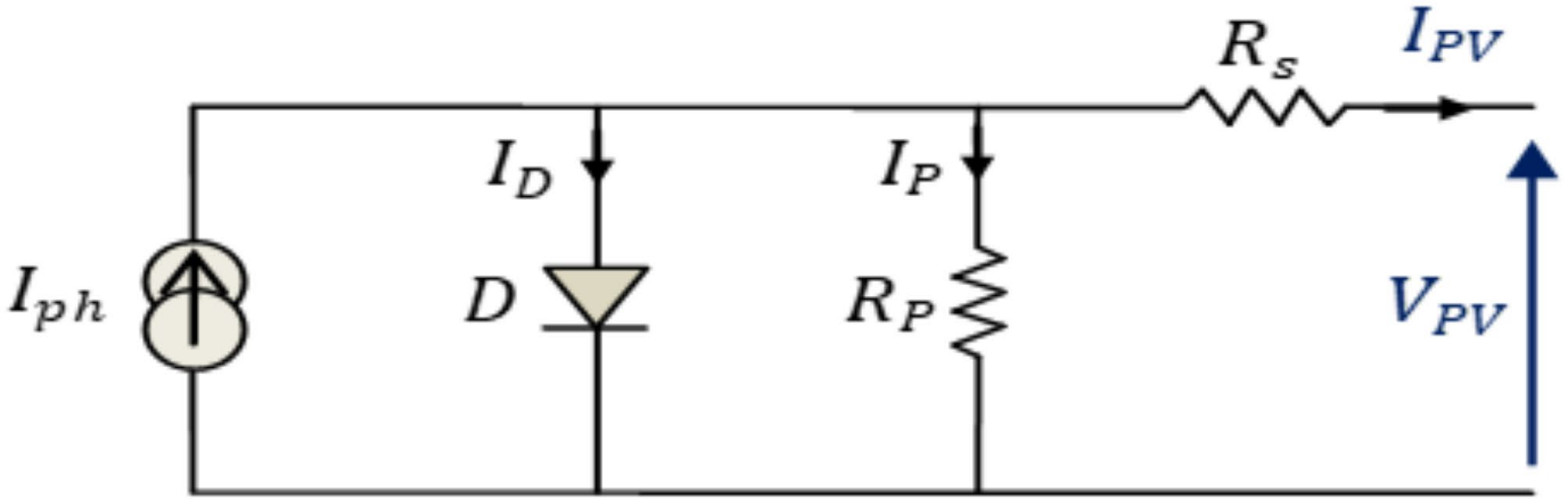
Simplified equivalent circuit of a photovoltaic cell.

Using semiconducting materials that demonstrate the PV effect, PV transforms light into electricity, making them valuable for applications like electricity generation. Figure 1 illustrates the model of a PV cell^32^. This solar cell’s output current can be written as a function of the currents flowing through the photovoltaic cell’s photodiode and its by-pass circuit:

1 $${I}_{o}={I}_{ph}-{I}_{d}-{I}_{sh}$$

The output current of a PV array is determined by the following equation:

2 $${I}_{o}={N}_{p}{I}_{ph}-{N}_{p}{I}_{rs}\left[{e}^{\left(\frac{q\left(v+{R}_{S}{.I}_{o}\right)}{A.k.T.{N}_{S}}\right)}-1\right]-{N}_{p}\left(\frac{q\left(v+{R}_{S}{I}_{o}\right)}{{N}_{S}.{R}_{Sh}}\right)$$

The equation below relates the solar irradiation to the photocurrent I_ph_ that is generated:

3 $${I}_{ph}=\left[{I}_{rs}+{k}_{i}\left(T-{T}_{r}\right)\right]\frac{S}{1000}$$

## Conventional (P&O) MPPT

**Fig. 2:**
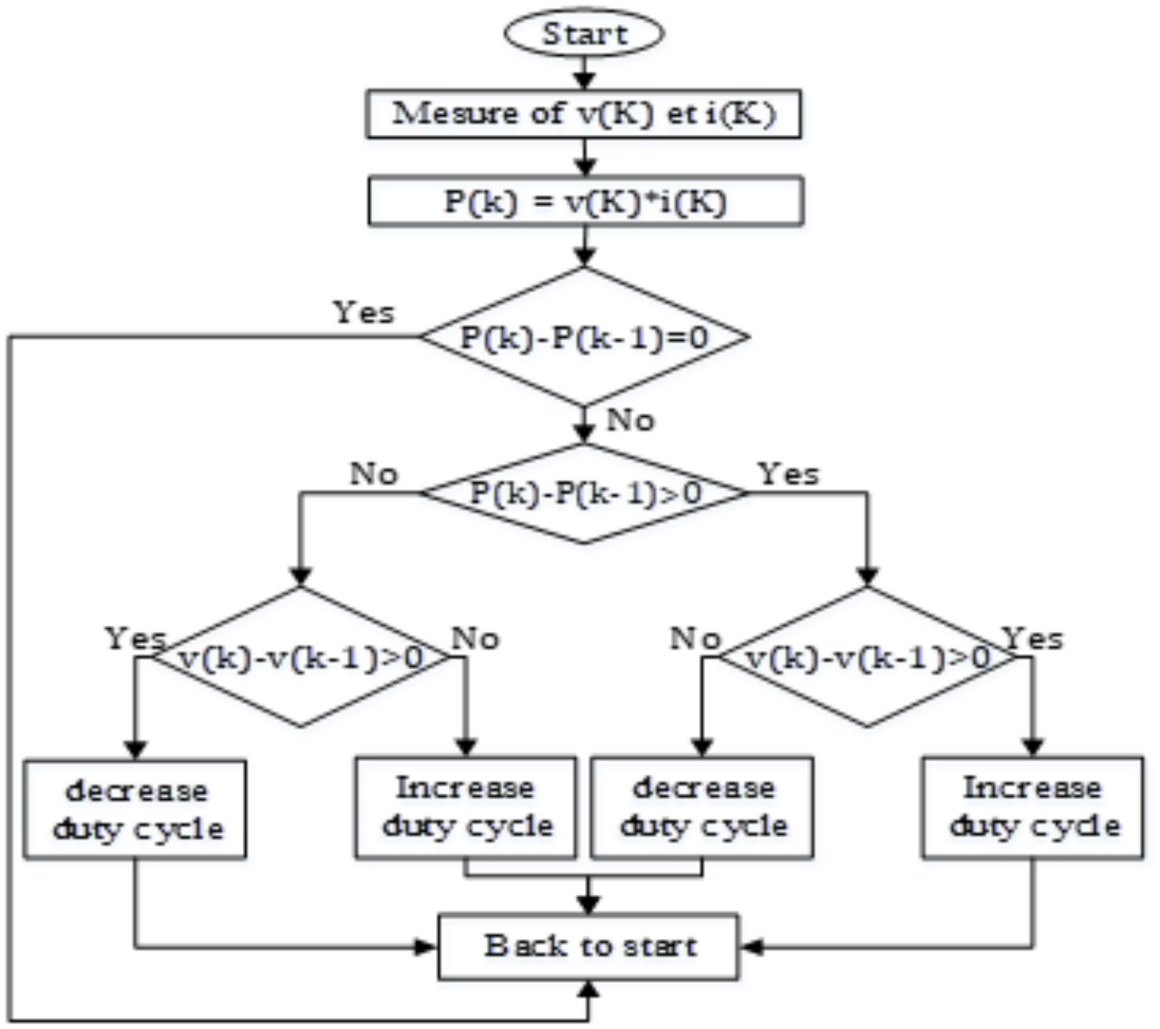
Illustrates the flowchart of the Perturb and Observe procedure, highlighting its simplicity and practicality in MPPT applications^33^.

PV are gaining prominence as a supplemental energy source, driven by the need to overcome their non-linear characteristics and low efficiency. Maximum PowerPoint (MPP) tracking is crucial in optimizing these systems, with Fig. 2. Flowchart illustrating the Conventional Perturb and Observe (P&O) Algorithm. Various strategies like Perturb and Observe (P&O), Incremental Conductance, and others proposed and implemented. P&O, a widely adopted technique, adjusts module operating points based on changes in output power polarity, enhancing efficiency through iterative voltage adjustments.

## Bio-inspired optimization algorithms: GWO AND WOA

GWO and WOA are examples of bio-inspired optimization algorithms that have garnered popularity as a result of their ability to effectively resolve a variety of optimization challenges. Inspired by the hunting and foraging behaviours of grey wolves and whales, these algorithms minimize dependency on random or user-defined parameters compared to other meta-heuristic methods. This makes them robust for real-world applications without requiring extensive methodological adjustments. Their application in Maximum Power Point Tracking (MPPT) for PV systems highlights their suitability and advantages in optimizing energy conversion processes efficiently. This section reviews the theoretical foundations and mathematical principles that enable GWO and WOA to achieve effective optimization outcomes^21^.

### Grey Wolf optimizer

The GWO method was formulated by Mirjalili et al.^34^, mimics the hierarchical structure and hunting strategies of grey wolf packs. The algorithm divides wolves into hierarchical groups: Alpha (α), Beta (β), Delta (δ), and Omega (ω). Alpha leads the pack, directing activities like hunting and migration. Beta assumes leadership if Alpha is incapacitated. Delta supports Beta and Alpha, while Omega represents other members^35^. GWO’s mathematical model incorporates three phases—surround, hunt, and attack prey—to efficiently guide search agents toward optimal solutions.

#### Encircling

The iteration begins (t = 1) in case the prey is found. Hence, the alpha (α), beta (β), and delta (δ) wolves guide the other search agents to track and ultimately surround the prey. This conduct of gray wolves is articulated as:

4 $$\overrightarrow{X}\left(\text{t}+1\right)=\overrightarrow{{X}_{p}}\left(t\right)+\overrightarrow{A}.\overrightarrow{D}$$

Where $\overrightarrow{X}$is The positions of the search agents (wolves), $\overrightarrow{{X}_{p}}$ Indicates the positions of the search agents (wolves) about the prey position, where II represents the iteration number. And $\overrightarrow{A}$ The vector represents the coefficients for (t + 1)^th^ iteration, in the case of $\overrightarrow{D}$, the other coefficient may be explained such as:

5 $$\overrightarrow{D}=\left|\overrightarrow{C},\overrightarrow{{X}_{p}}\left(t\right)-\overrightarrow{X}\left(t\right)\right|$$

The $\overrightarrow{A}$ and $\overrightarrow{C}$ parameteric group of manipulating variables with stochastic values $\overrightarrow{{r}_{1}}$ and $\overrightarrow{{r}_{2}}$ It ought to be represented using mathematical notation as:

6 $$\overrightarrow{A}=2\overrightarrow{a}.\overrightarrow{{r}_{1}}-\overrightarrow{a}$$

7 $$\overrightarrow{C}=2.\overrightarrow{{r}_{2}}$$

Where components of $\overrightarrow{a}$are linearly decreasing from 2 to 0 throughout the iterations $\overrightarrow{{r}_{1}}$,$\overrightarrow{{r}_{2}}$are random vectors in [0,1].

#### Prey hunting

During hunting, grey wolves strategically reposition each group relative to prey locations. Alpha, Beta, and Delta guide Omega to potential hiding places, adapting their movements to optimize hunting efficiency. Equations describe how locations are updated in subsequent rounds, reflecting the pack’s coordinated pursuit and adaptation to maximize hunting success.

8 $$\begin{array}{c}\overrightarrow{{D}_{\alpha}}=\left|\overrightarrow{{C}_{1}^{t}}.\overrightarrow{{X}_{\alpha}^{t}}-{X}^{t}\right|\\ \overrightarrow{{D}_{\beta}}=\left|\overrightarrow{{C}_{1}^{t}}.\overrightarrow{{X}_{\beta}^{t}}-{X}^{t}\right|\\ \overrightarrow{{D}_{\delta}}=\left|\overrightarrow{{C}_{1}^{t}}.\overrightarrow{{X}_{\delta}^{t}}-{X}^{t}\right|\end{array}$$

9 $$\begin{array}{c}\overrightarrow{{X}_{1}^{t}}=\overrightarrow{{X}_{\alpha}^{t}}-{A}_{1}^{t}.\overrightarrow{{D}_{\alpha}^{t}}\\ \overrightarrow{{X}_{2}^{t}}=\overrightarrow{{X}_{\beta}^{t}}-{A}_{2}^{t}.\overrightarrow{{D}_{\beta}^{t}}\\ \overrightarrow{{X}_{3}^{t}}=\overrightarrow{{X}_{\beta}^{t}}-{A}_{3}^{t}.\overrightarrow{{D}_{\beta}^{t}},\end{array}$$

10 $${X}^{\text{t}+1}=\frac{{X}_{1}^{t}+{\text{X}}_{2}^{t}+{\text{X}}_{3}^{t}}{3}$$

#### Attacking the prey

**Fig. 3:**
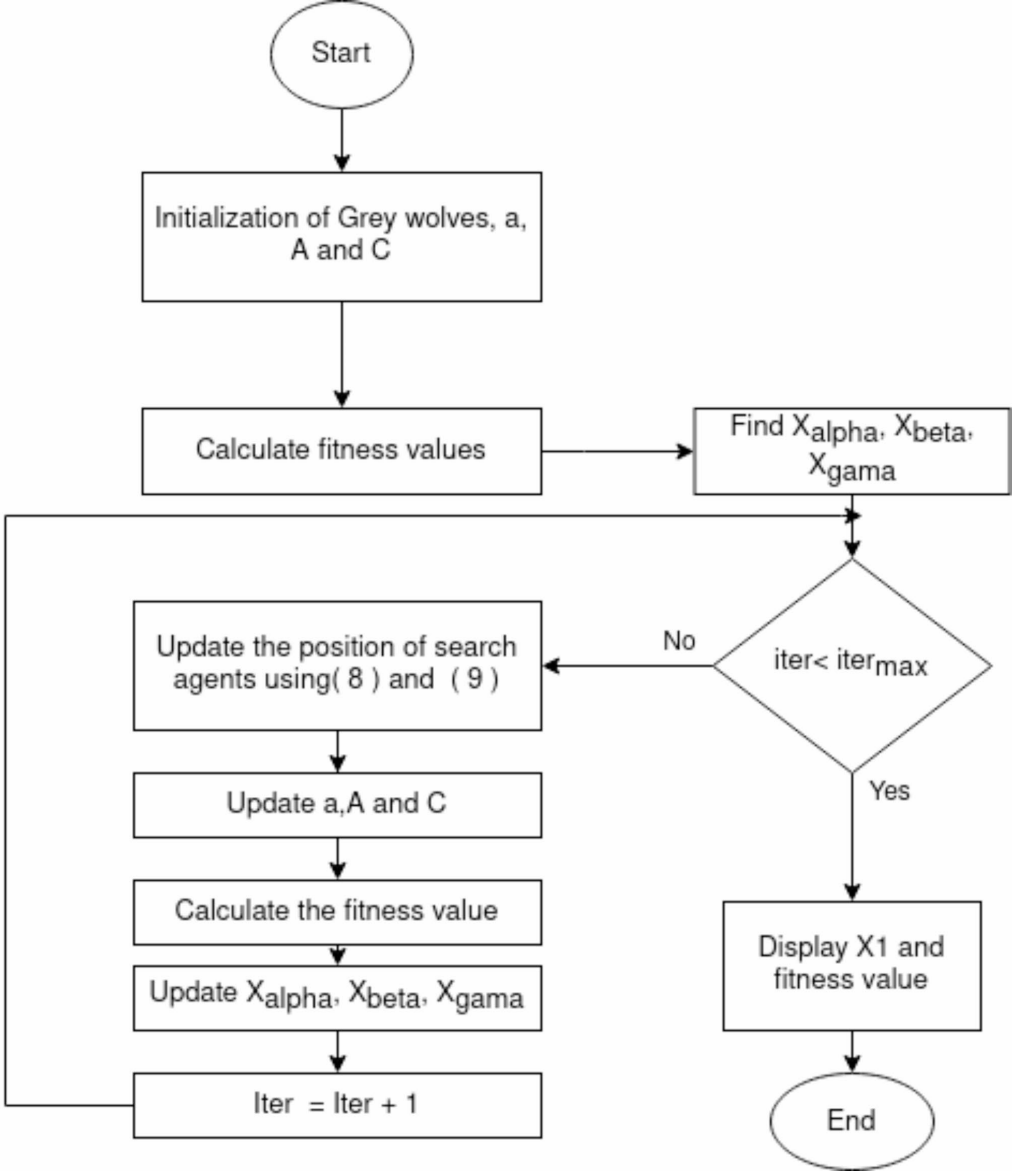
Diagram illustrating the GWO algorithm’s flow.

A parameter guides the attacking process. The vector $\overrightarrow{A}$ is modified by$\overrightarrow{a}$ to direct omega type wolves towards or in other direction from the prey (solution). It’s exciting to think about what could happen if a group of wolves ventured out to explore new territories in space. Otherwise, they approach dominants, leading omega wolves to follow and explore a larger search space. $\overrightarrow{D}=\left|\overrightarrow{C},\overrightarrow{{X}_{p}}\left(t\right)-\overrightarrow{X}\left(t\right)\right|$ Continuing the trend, the value gradually decreases from 2 to 0 throughout iterations:

11 $$\overrightarrow{a}=2\left(1-t/N\right)$$

where t is the number of iterations that are currently in progress and N is the total number of iterations. Figure 3 illustrates the following diagram shows the basic stages involved in the GWO:

### Whale optimization algorithm (WOA)

**Fig. 4:**
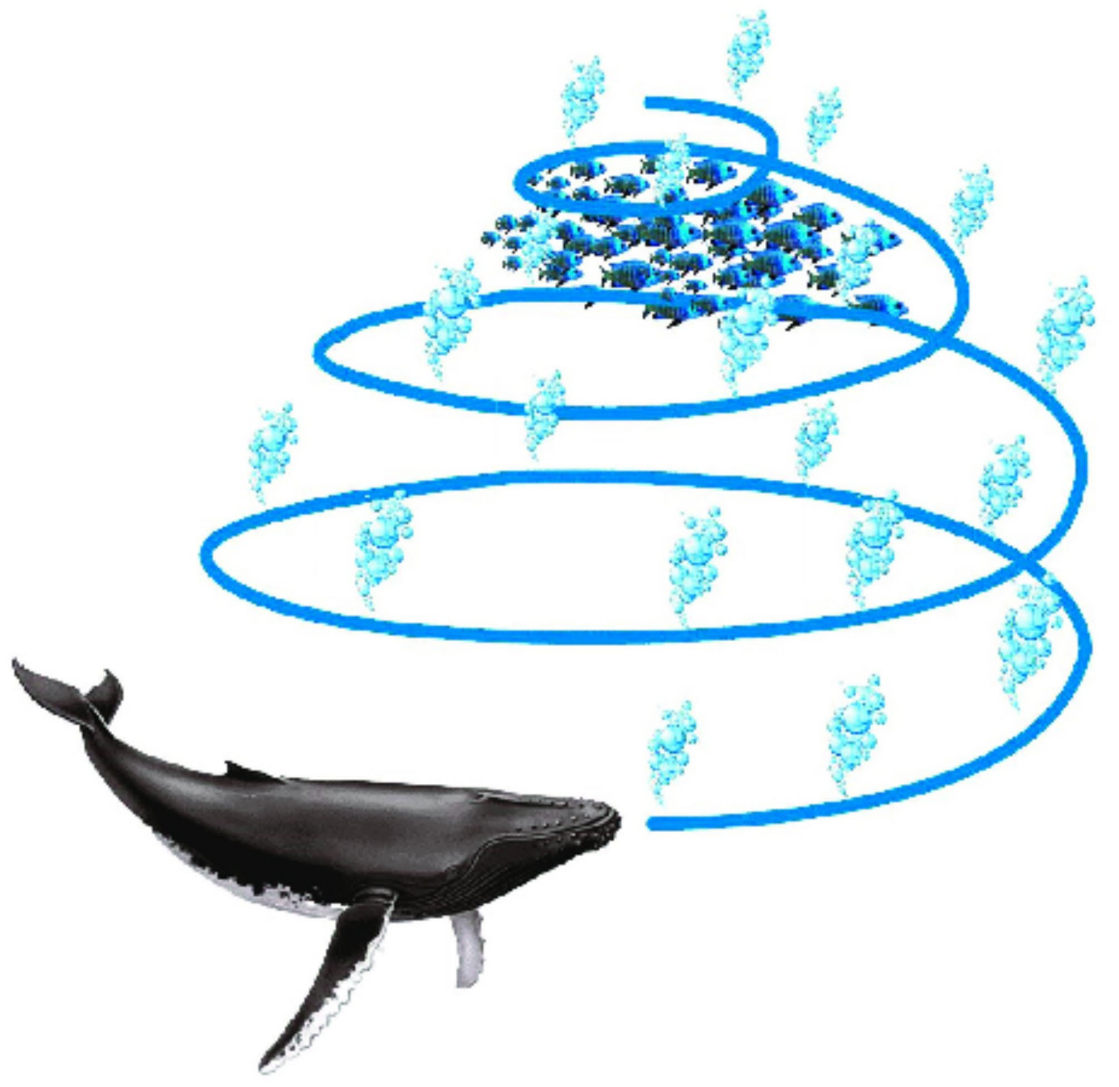
The feeding behaviour of humpback whales is known as “bubble-net feeding.”

The Whale Optimization Algorithm (WOA) was proposed by Mirjalili et al.^36^ draws inspiration from the cooperative feeding behaviour of humpback whales. Specifically, WOA mimics the bubble-net feeding strategy where whales encircle prey in a coordinated manner. This behaviour is illustrated in Fig. 4, showcasing the collaborative nature of humpback whales during feeding. The algorithm is structured into three phases: surrounding the prey, initiating an attack with a bubble net, and exploring the surroundings for optimal solutions. The first two phases focus on exploiting known solutions, while the third phase emphasizes exploration to discover new potential solutions. Mathematical principles underpinning WOA’s model guide these phases, facilitating efficient optimization processes.

#### Exploitation phase (encircling prey, a bubble-net attacking)

Mathematical Eqs. (12) and (13) can be employed to simulate the hunting behaviour of humpback whales, as described in^29^.

12 $$\overrightarrow{D}=\left|\overrightarrow{C}.\overrightarrow{{X}^{{\prime}}}\left(t\right)-\overrightarrow{X}\left(t\right)\right|$$

13 $$\overrightarrow{X}\left(\text{t}+1\right)=\overrightarrow{{\text{X}}^{\prime}}\left(t\right)-\overrightarrow{\left(A\right)}.\overrightarrow{D}$$

where ***t*** denotes the current iteration, *X’* represents the position vector, and X signifies the best solution acquired up to the present iteration. A and C are coefficient vectors determined according to the equations specified in (14) and (15).

14 $$\overrightarrow{A}=2\overrightarrow{a}.\overrightarrow{r}-\overrightarrow{a}$$

15 $$\overrightarrow{C}=2.\overrightarrow{r}$$

In the Whale Optimization Algorithm (WOA), the exploration and exploitation phases are managed by the parameter ‘a’, which linearly decreases from a maximum value (a²) to zero over iterations. Random vector ‘a’ is uniformly distributed in [0, 1]. Changes in parameters ‘r’ and ‘values’ influence solution positions, guiding search agents towards optimal solutions. Humpback whales’ hunting strategy involves a contracting encircling mechanism and a spiral path towards prey, as defined in Eq. (16) to optimize search efficiency.

16 $${\text{a}} = 2-t\frac{2}{{\text{MaxIter}}}$$

In this case, ‘t’ represents the current number of iterations number, whereas ‘MaxIter’ indicates the greatest allowable count of iterations. The proximity among the current solution as well as the optimal point is assessed by employing Eq. (17) to calculate the spiral-shaped trajectory.

17 $$\overrightarrow{X}(\text{t}+1)={\text{D}}^{\prime}{\text{e}}^{\text{bl}}.{\cos}\left(2\varPi l\right)+\overrightarrow{{\text{X}}^\prime}(t)$$

The Whale Optimization Algorithm (WOA) uses the distance metric ${\text{D}}^{\prime}=\left|\overrightarrow{{\text{X}}^{\prime}}(t)-\overrightarrow{X}(t)\right|$ to gauge the distance between the ith whale and the current best solution (prey). It introduces a random coefficient ‘p’ ranging from 0 to 1, which probabilistically selects between two strategies during optimization rounds: a spiral-shaped route and a shrinking encircling method, each with a 50% chance. If ‘p’ is less than 0.5, the shrinking encircling method is employed to update positions.

#### Search of prey

In the Whale Optimization Algorithm (WOA), the construction of bubble networks incorporates a probabilistic search for prey, enhancing its exploration phase. This phase involves adjusting the coefficient ‘A’, which can vary within the interval [-1, 1]. Random updates to the distance data *D* occur during this phase, enabling whales to deviate from the initially identified optimum, thereby enhancing the algorithm’s capability for global search.

18 $$\overrightarrow{D}=\left|\overrightarrow{C}.\overrightarrow{{X}_{{\text{rand}}}}-\overrightarrow{X}\right|$$

19 $$\overrightarrow{X}\left(\text{t}+1\right)=\overrightarrow{{X}_{{\text{rand}}}}-\overrightarrow{A}.\overrightarrow{D}$$

**Fig. 5:**
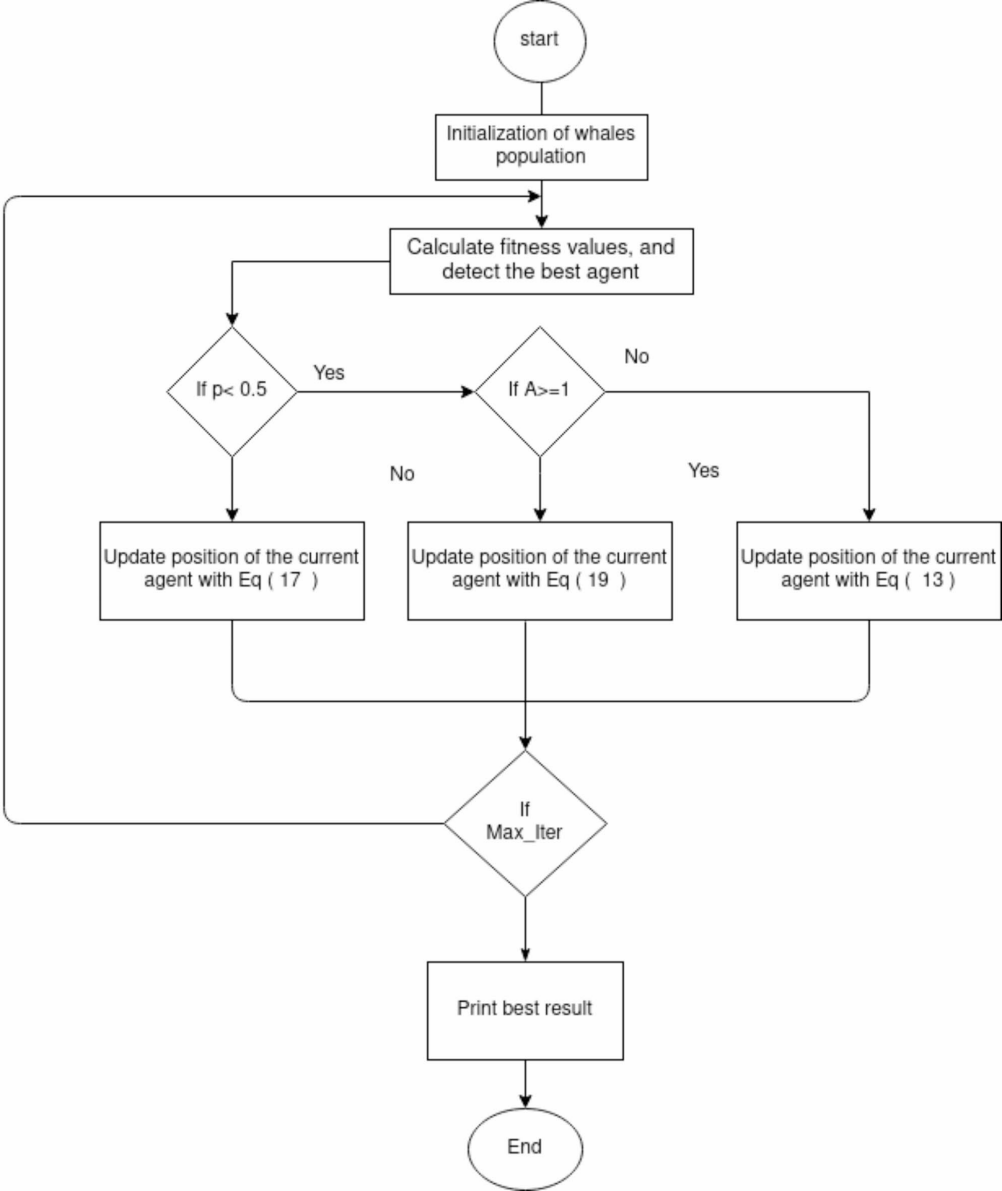
Whale optimization algorithm (WOA) flowchart.

Where$:{I}_{\alpha}={I}_{pk}-{I}_{d}-$, $\overrightarrow{{X}_{rand}}$ It shows the randomly selected geographical information of a whale from the present run. The WOA approach’s flowchart is shown in Fig. 5.

## MPPT algorithm with variable step size using GWO and WON

### System implementation

Traditional MPPT algorithms with fixed step sizes excel in performance but suffer from slow convergence, oscillations near MPP, and challenges in adapting to sudden atmospheric changes. Larger step sizes offer faster tracking but introduce steady-state oscillations, while smaller steps reduce oscillations at the expense of slower dynamics. Recent advancements in PV array optimization introduce variable step size algorithms that adjust step sizes based on PV array characteristics, balancing dynamics and oscillations.

**Fig. 6:**
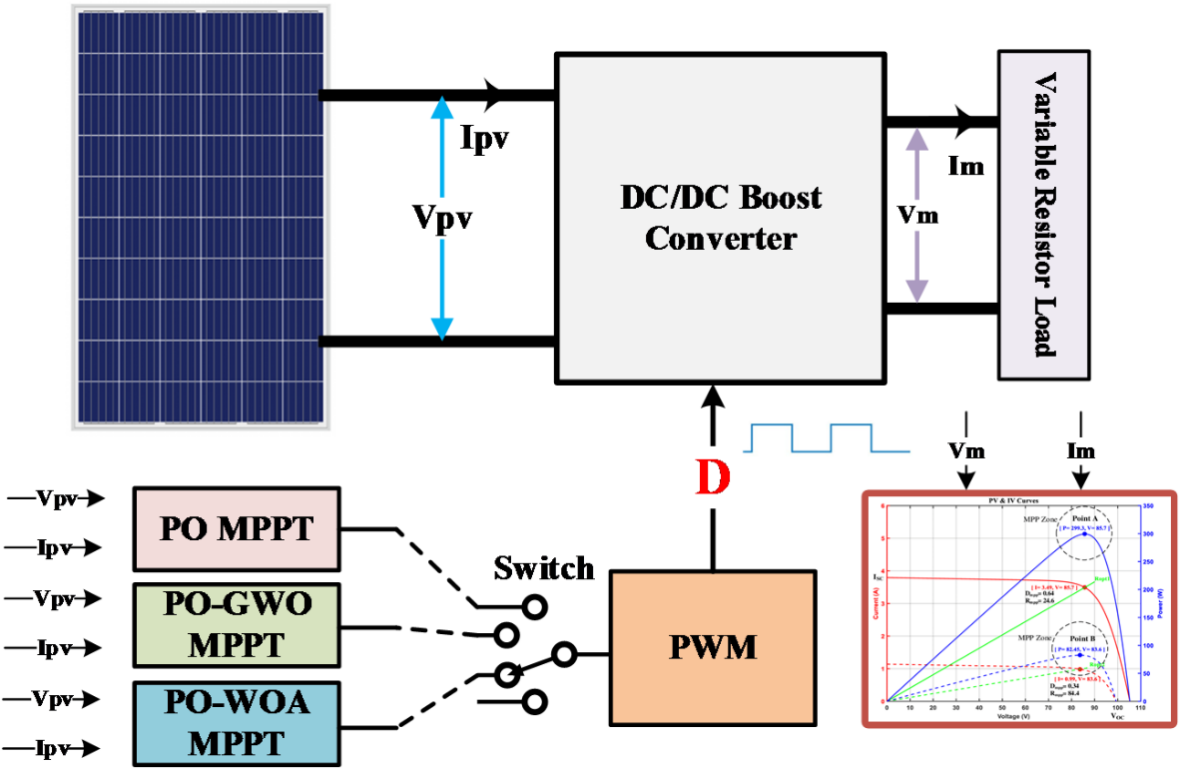
Variable step size MPPT methods schematic diagram.

**Fig. 7:**
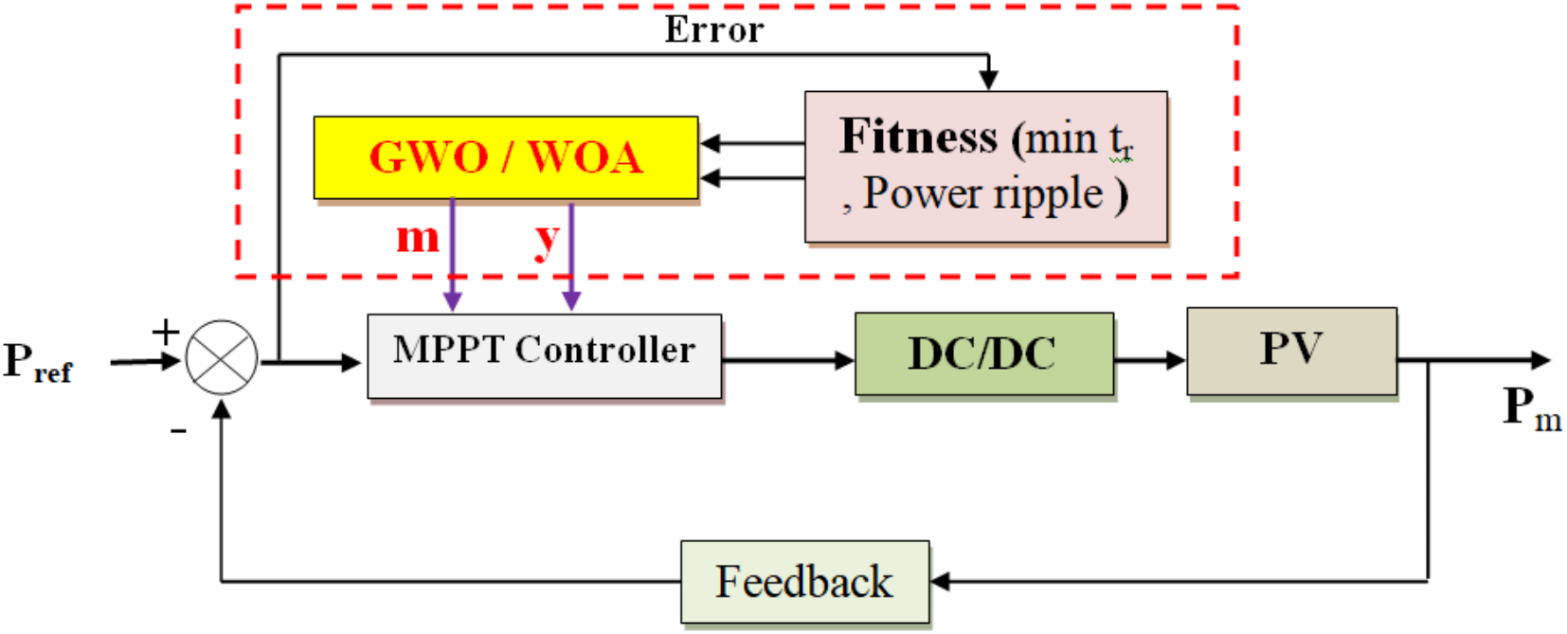
Optimization of step size using GWO and WOA.

This work introduces a novel MPPT method that utilizes a configurable step size for improved implementation ease, faster response times, and reduced oscillations. Figure 6 illustrates variable step size MPPT methods schematic diagram, utilizing Eq. (20) to adjust duty cycle D(k) based on the scaling factor *N* and PV output power *dP*. Integrating GWO/WOA for step size tuning enhances MPPT performance under typical PV conditions, as depicted in Fig. 7’s system block diagram.

20 $$D\left(k\right)=D\left(k-1\right)\pm{\text{N}}{*}dP$$

D(k): the duty cycle and its corresponding coefficient.

N: _ The scaling factor was modified during the sampling period to control the step size.

dP: the PV array output power derivation.

### Objective function

the objective function plays a critical role in optimizing any problem. This study emphasizes precise outcomes by integrating evaluation criteria: Integral Square Error (ISE) for ripple evaluation and overshoot criteria to enhance system response time^37^. ISE is calculated as :

21 $$ISE={\int}_{0}^{\tau}{\left({P}_{ref}-{P}_{out}\right)}^{2}dt$$

while overshoot is defined as:

22 $$Overshoot=max\left({P}_{out}\right)-Pref$$

The fitness function combines these metrics:

23 $$F=\alpha.ISE+\beta.Overshoot$$

with and *β* (both 0.5 in this study) balancing between objectives.

## Results and discussion

The Solarex MSX-60 PV module consists of 36 solar cells, was chosen for simulations and analysis of the proposed method^13^. Table 1 displays the module’s electrical characteristics when S = 1000 W/m^2^ and T = 25 °C as input. As a bonus,

**Table 1:** Solar power system parameters / msx − 60 (1kw/m², 25 °C).

Characteristics	MSX-60
P_m_: Maximum Power	60 W
V_m_: Voltage Pm	17.1 V
I_m_: Current at Pm	3.5 A
I_sc_: Short Circuit Current	3.8 A
V_oc_: Open Circuit voltage	21.1 V

Five steps of irradiation signals were simulated using MATLAB/Simulink, following the specifications outlined in Table 2. These simulations aim to demonstrate the effectiveness of our proposals in handling sudden changes in irradiation caused by weather fluctuations or passing clouds. This was undertaken to compare and contrast the results against those obtained using the standard P&O algorithm.

**Table 2:** Signals for test patterns.

Radiation (W/m^2^ )	t (s)
1000	0–0,5
600	0,5–1
800	1–1,5
600	1,5–2
1000	2–2,5

GWO and WOA offer advantages due to their minimal user-specified parameters, which are discussed in the bio-inspired algorithms section. Both algorithms share three primary parameters: iterations, agents, and optimized variables (representing step sizes m and y for the test model). Table 3 details the specific parameter metrics for each algorithm.

**Table 3:** Setup parameters for WAO and WAO.

Description	Parameters	
	GWO	WAO
Number of search agents	10	10
Maximum number of iterations	20	20
Number of variables	2	2

**Fig. 8:**
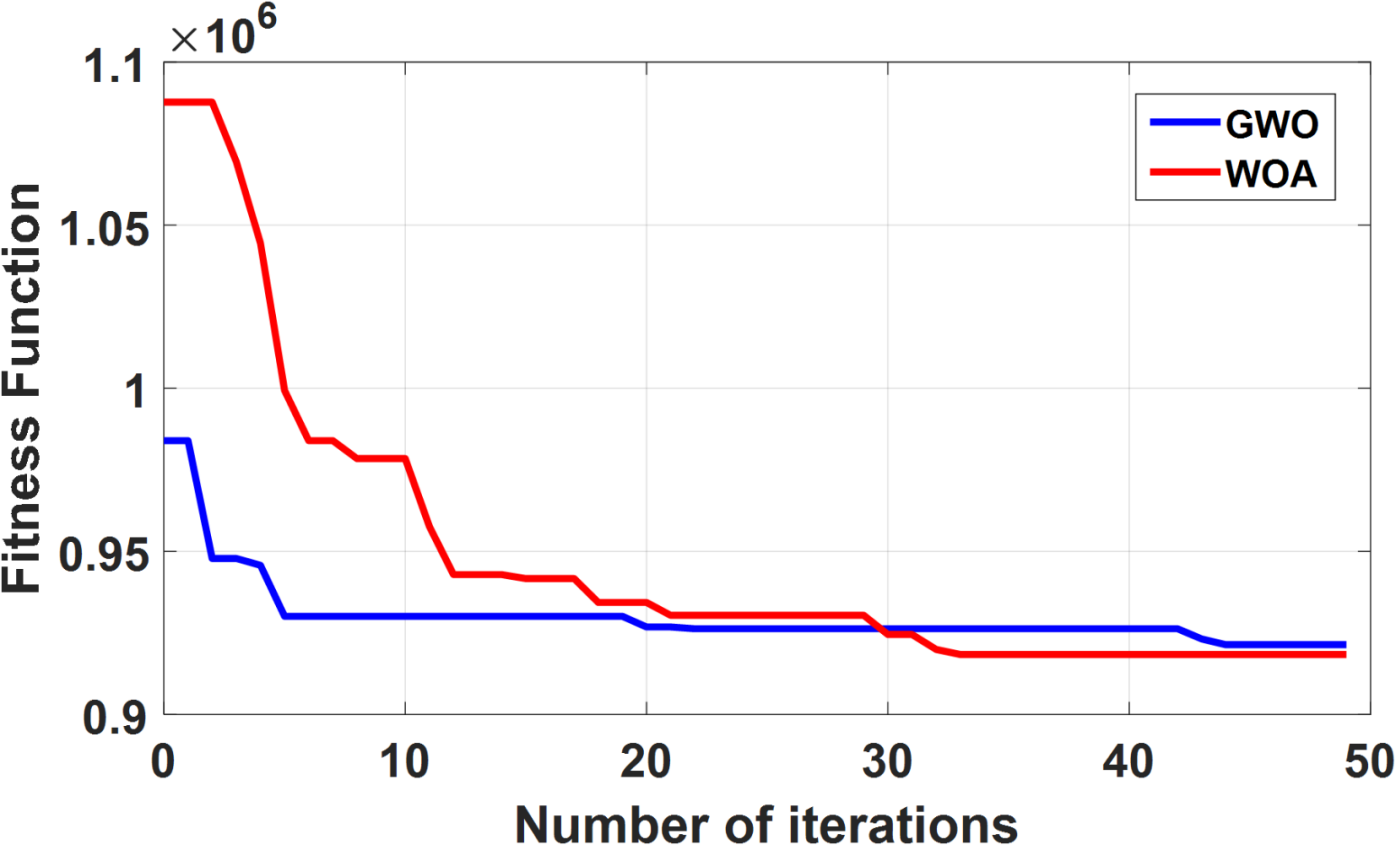
Fitness value evolution curves.

In a comparative analysis, WOA demonstrates superior performance over GWO in minimizing the fitness function (Fig. 8). Owing to its balanced exploitation-exploration strategy, WOA achieves enhanced results in reducing overshoot and improving the time of response. In contrast, GWO’s hierarchical search approach initially yields lower fitness values but adversely affects ripple control in the model.

Table 4 summarizes four separate runs with the same global objective and recommended strategies. It highlights the results for ripple, overshoot, and response time in bold for easy reference, guiding the subsequent stages of our research.

**Table 4:** The optimum set of controllers gains.

Algorithm		m	y
GWO	Test 1	-0.015	-0.001
	**Test 2**	**-0.0016**	-0.000
	Test 3	-0.019	-0.0042
	Test 4	-0.018	-0.0052
WOA	Test 1	-0.016	-0.0049
	Test 2	-0.02	-0.0012
	**Test 3**	**-0.005**	**-0.0016**
	Test 4	-0.0180	-0.0013

**Fig. 9:**
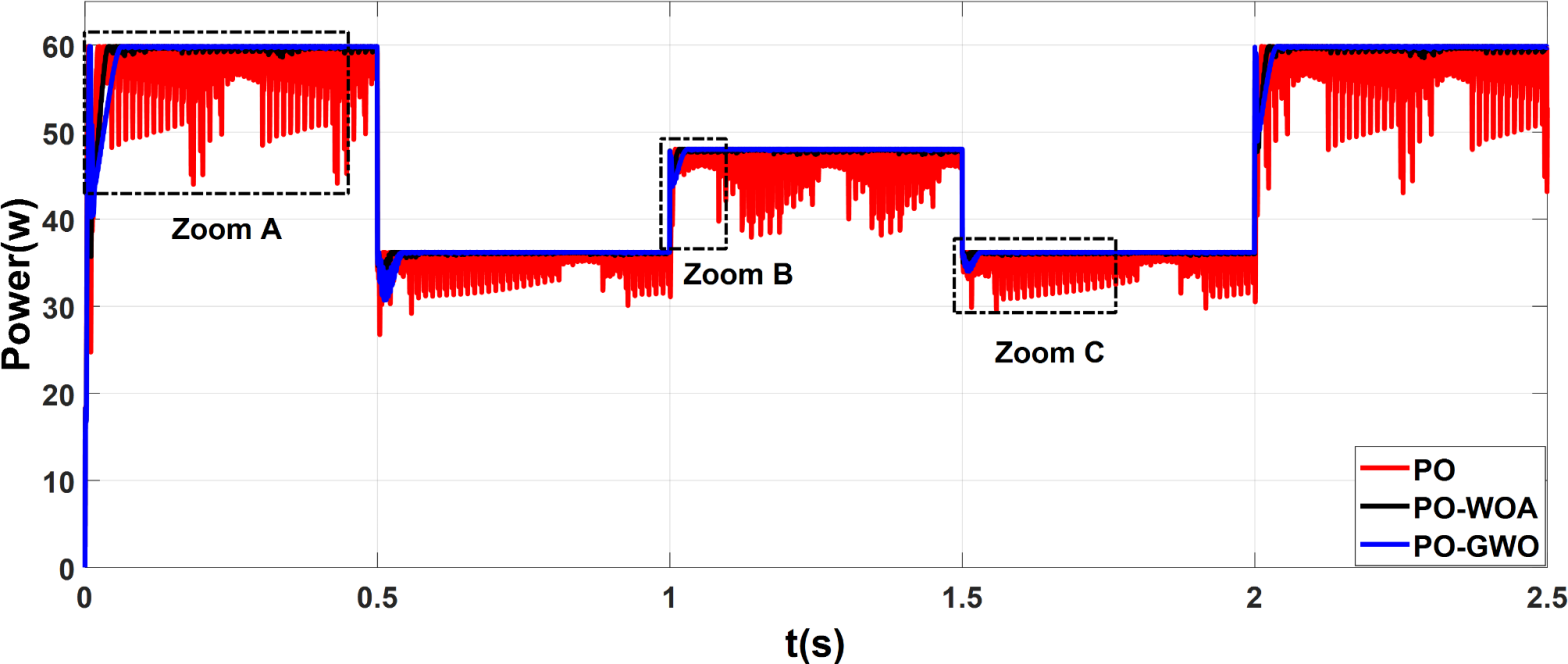
The output power of MPPT using PO, PO-GWO and PO-WOA.

**Fig. 10:**
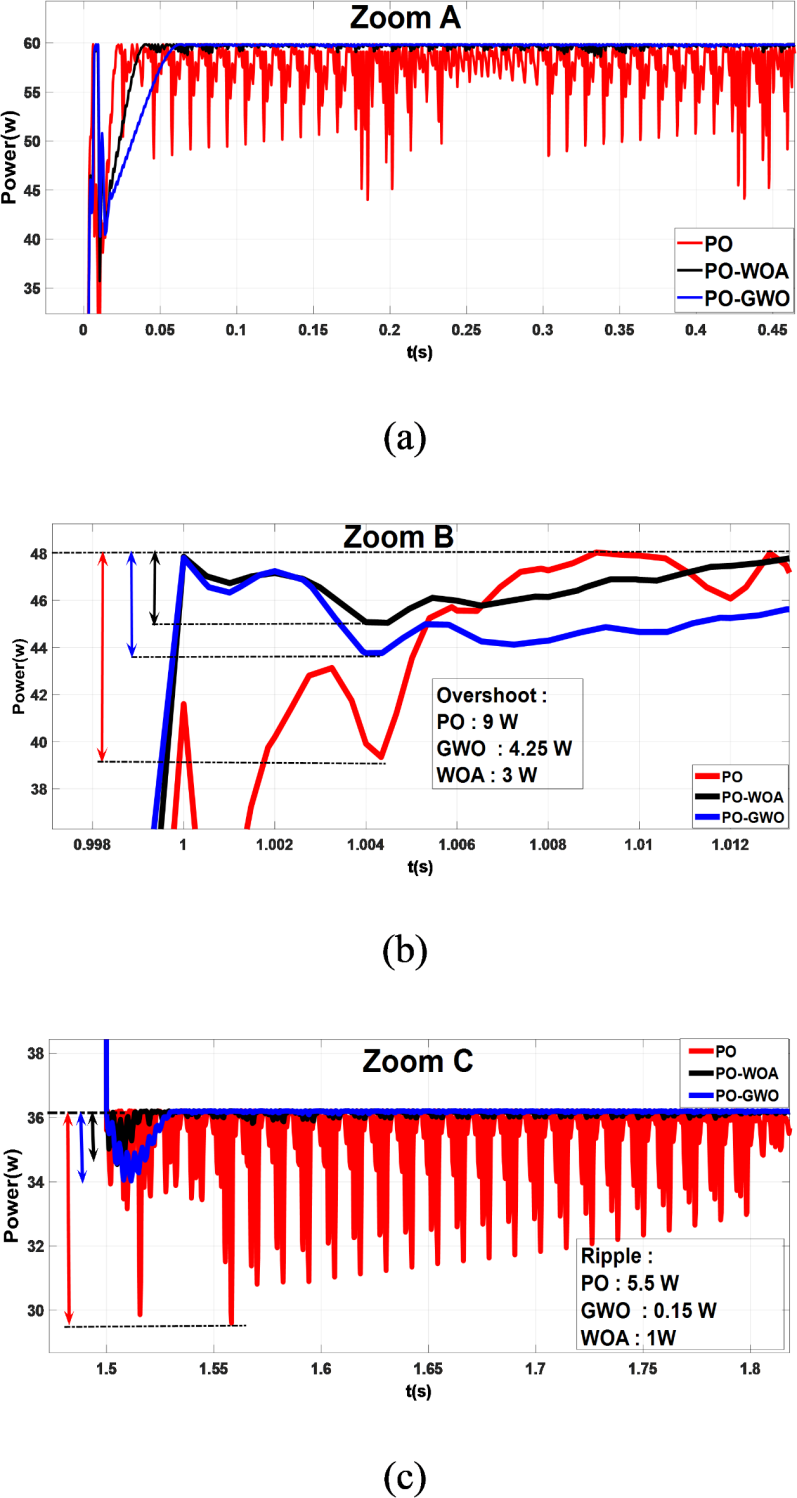
Comparative analysis of MPPT tracking (**a**), power overshoot (**b**), and power ripple (**c**) for the proposed algorithms.

**Fig. 11:**
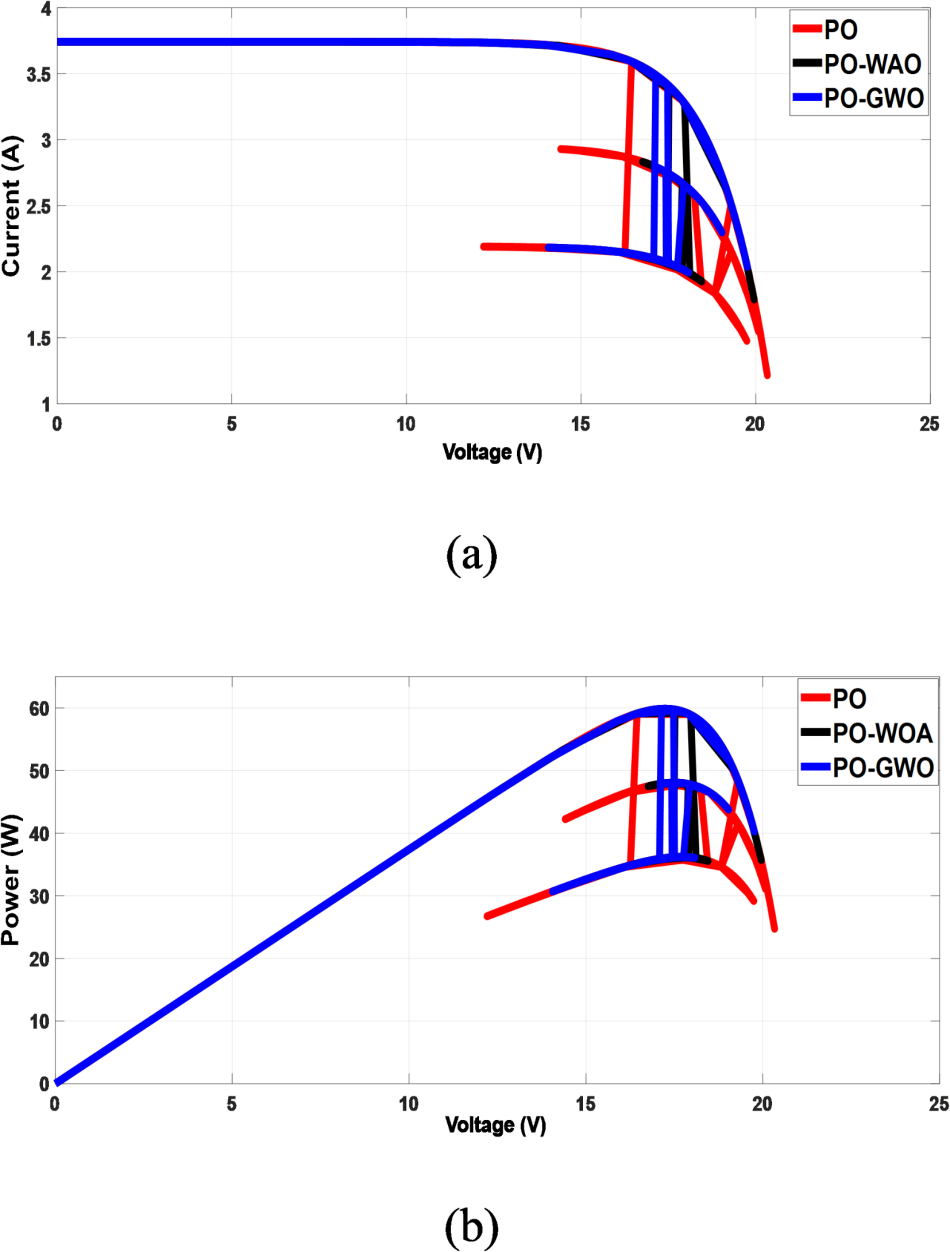
Comparison of MPPT: fixed step (PO) vs. variable step (GWO/WOA). (**a**) I-V characteristics, (**b**) P-V characteristics.

A comparison was conducted between MPPT algorithms using fixed step sizes and those employing variable step sizes to assess the efficiency of the proposed GWO MPPT and WOA MPPT approaches. The results demonstrate improvements in three performance metrics: tracking accuracy and ripple reduction. Figures 9 and 10 show the outcomes of offline training steps for both GWO and WOA, while Fig. 11 presents the results achieved using optimized fixed and variable step size MPPT methods.

Simulation results demonstrate that both fixed and variable step-size MPPT algorithms effectively track the MPP under varying irradiance conditions, closely aligning measured power with the theoretical values predicted by irradiance (Fig. 11). However, significant differences in performance metrics, particularly in overshoot and ripple, highlight the advantages of the proposed variable step-size methods.

The fixed step-size PO MPPT controllers exhibit higher power peak overshoot of 9 W during rapid meteorological changes, while the variable step-size methods show significant improvements. The GWO algorithm reduces overshoot to 4.5 W (50% reduction), and the WOA algorithm further reduces it to 3 W (67% reduction), as illustrated in Fig. 10b. Improved overshoot control reduces energy loss and stress on components, contributing to the overall reliability and longevity of the PV system. In terms of ripple, the proposed GWO and WOA methods demonstrate superior performance. The fixed step-size PO controller produces a ripple of 5.5 W, whereas the GWO algorithm reduces it to 1 W (82% reduction), and the WOA algorithm achieves an even greater reduction to 0.15 W (98% reduction), as shown in Fig. 10c. Significant ripple reduction ensures smoother system operation, which is critical for improving the efficiency and stability of the power supply under dynamic environmental conditions.

The performance improvements achieved by the proposed GWO and WOA algorithms, compared to the baseline PO method, are summarized in Table 5. This table highlights the reductions in ripple and overshoot achieved by the variable step-size MPPT algorithms, along with their corresponding improvement percentages and practical implications.

**Table 5:** Comparison of ripple and overshoot performance for PO, GWO, and WOA MPPT algorithms.

Performance metric	Baseline (PO)	PO-GWO	PO-WOA	Improvement: Reduction ratio (%)	
				PO-GWO	PO-WOA
Ripple (W)	5.5	1	0.15	82%	98%
Overshoot (W)	9	4.5	3	50%	67%

## Real data investigation

The investigation was conducted at the Ain El Melh photovoltaic (PV) station in Algeria, located at a latitude of 34.859453°N and a longitude of 4.201124°E. Data was collected on June 21, 2023, to evaluate the efficiency, reliability, and applicability of the proposed strategy throughout the day. The PV module was examined over 11 h, from 8:00 AM to 6:00 PM, with irradiance measurements taken at one-hour intervals. To ensure accurate monitoring of solar radiation and other meteorological parameters, a weather station was installed adjacent to the PV arrays. This station is equipped with an extensive suite of sensors designed to capture essential climatic data. Two pyrometers measure both direct and diffuse solar radiation, providing critical insights into the solar energy received. Ground humidity levels are monitored by a dedicated sensor, while a TDZ02-1 rainfall sensor quantifies precipitation. This robust monitoring infrastructure enables the acquisition of accurate meteorological data, supporting energy management, solar energy optimization, and weather forecasting.

**Fig. 12:**
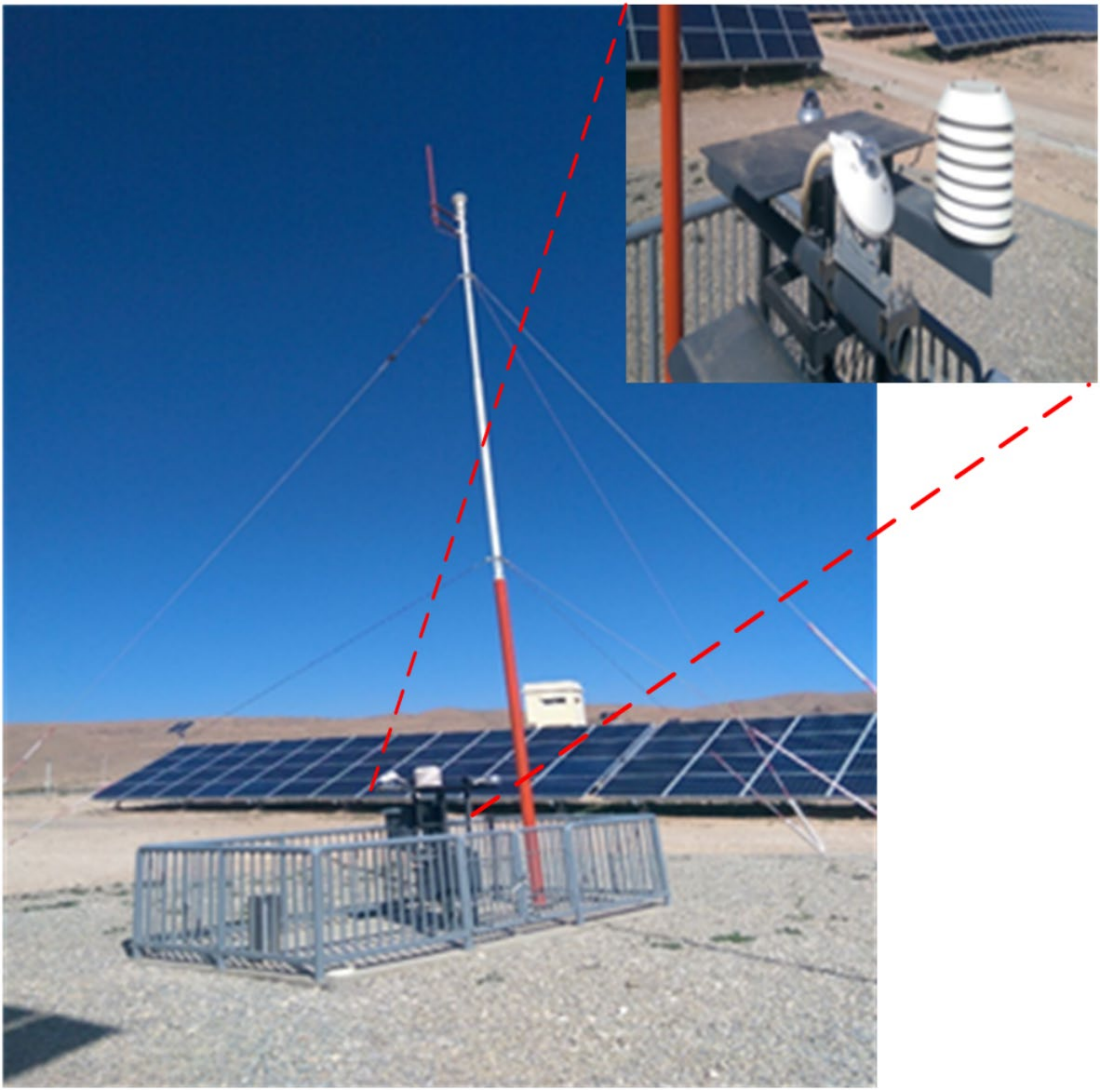
Overview of the weather station components utilized.

**Fig. 13:**
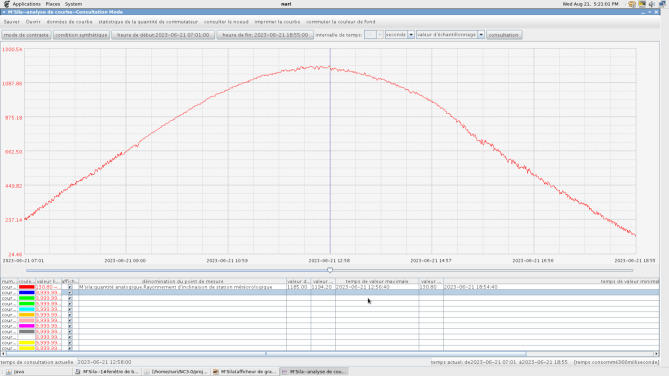
Irradiation levels data for June 21, 2023, at Ain Elmelh PV station in Algeria.

Figure 12 provides an overview of the Ain El Melh PV station and its weather monitoring components, while Fig. 13 illustrates the experimental irradiance data recorded during the study.

**Fig. 14:**
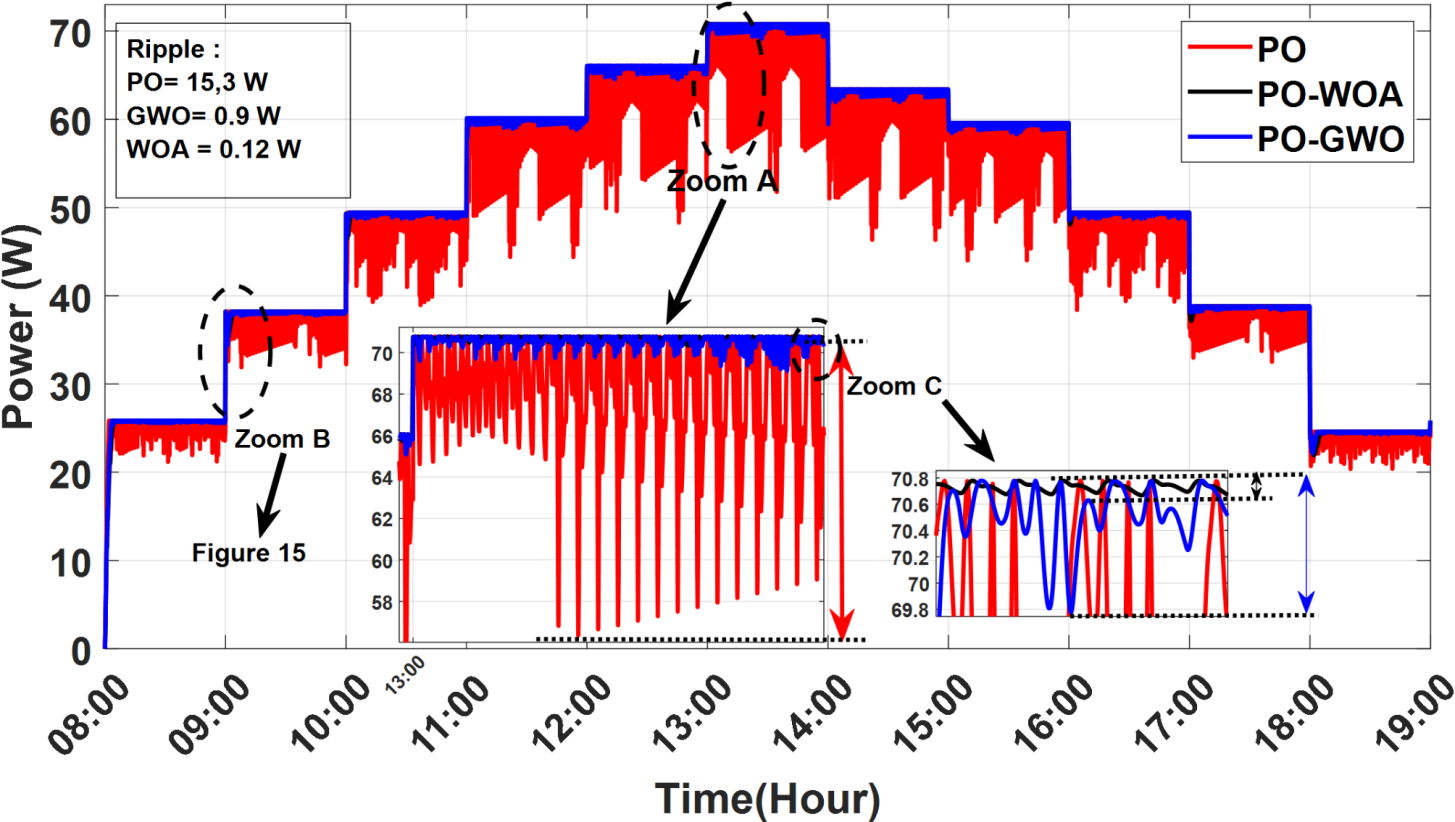
Output power of MPPT using PO, PO-GWO and PO-WOA on June 21, 2023.

**Fig. 15:**
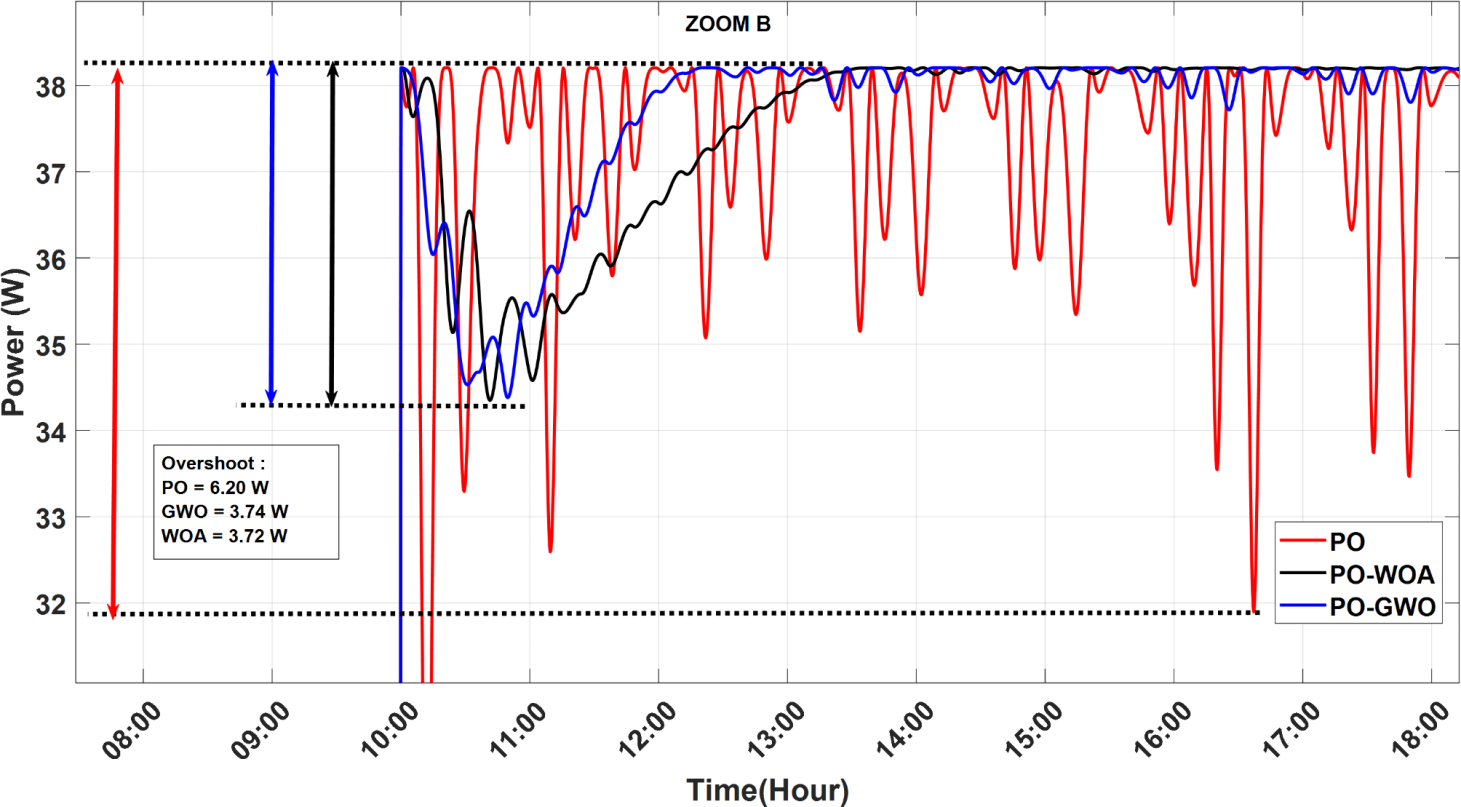
Comparative analysis of power overshoot in MPPT tracking for PO, GWO, and WOA algorithms.

**Fig. 16:**
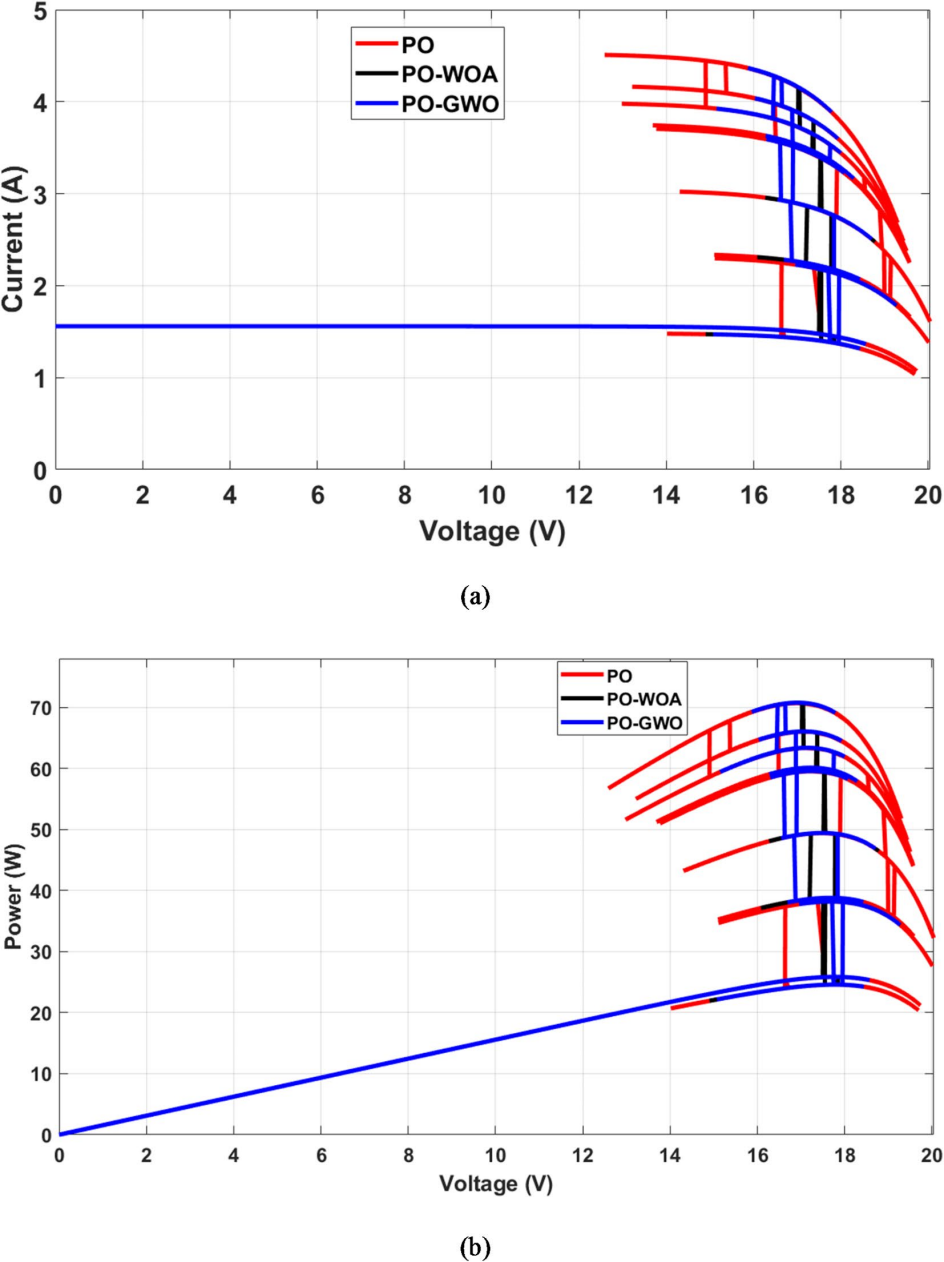
MPPT performance comparison: fixed step (PO) vs. variable step (GWO/WOA). (**a**) I-V characteristics, (**b**) P-V characteristics.

A comparative analysis was carried out to evaluate the performance of MPPT algorithms utilizing fixed step sizes against those employing variable step sizes, with a focus on the efficiency of the proposed GWO and WOA MPPT strategies. The findings reveal significant enhancements in key performance metrics, including improved tracking accuracy and reduced power ripples. Figures 14, 15 and 16 illustrate the outcomes achieved with both fixed and optimized variable step-size MPPT methods, highlighting the advantages of the proposed approaches.

Results demonstrate that both fixed and variable step-size MPPT algorithms effectively track the maximum power point under varying irradiance conditions throughout the day on June 21, 2023, from 8:00 AM to 6:00 PM. However, significant differences emerge in both overshoot and ripple performance. The fixed step-size PO MPPT controller exhibits a substantially higher power peak overshoot of 6.2 W during rapid meteorological changes, compared to the variable step-size algorithms. The GWO algorithm reduces overshoot to 3.74 W (39.6% reduction), while the WOA algorithm achieves a slightly better result with 3.72 W (40% reduction). In terms of ripple, the fixed step-size PO controller produces a ripple of 15.3 W, whereas the variable step-size algorithms show a significant reduction. The GWO algorithm reduces ripple to 0.9 W (94% reduction), and the WOA algorithm achieves an even better result of 0.12 W (99% reduction). This reduction in ripple ensures smoother power output, which is crucial for improving system efficiency and stability under dynamic environmental conditions. Figure 16 highlights the instability of the fixed step-size PO MPPT approach compared to the stability achieved by the GWO and WOA variable step-size MPPT strategies. These findings underscore the superior performance of the GWO and WOA algorithms in optimizing solar PV system efficiency, particularly in mitigating both ripple and overshoot during rapid environmental changes.

The performance comparison of ripple and overshoot for the PO, GWO, and WOA MPPT algorithms throughout the day on June 21, 2023, is summarized in Table 6, highlighting the significant improvements achieved by the variable step-size algorithms in both ripple reduction and overshoot control.

**Table 6:** Comparison of ripple and overshoot performance for PO, GWO, and WOA MPPT algorithms throughout the day (June 21, 2023).

Performance metric	Baseline (PO)	PO-GWO	PO-WOA	Improvement: Reduction ratio (%)	
				PO-GWO	PO-WOA
Ripple (W)	15.3	0.9	0.12	94%	99%
Overshoot (W)	6.2	3.74	3.72	39.6%	40%

## Comparative analysis of MPPT algorithm performance metrics

Table 7 presents a comparative analysis of the performance metrics of three MPPT algorithms—PO, PO-GWO, and PO-WOA—under both simulated irradiance profiles and real irradiation data recorded on June 21, 2023. The key metrics evaluated include average efficiency, average power loss, and average response time.

### Average efficiency

Efficiency is a critical metric for evaluating MPPT performance. The results demonstrate a marked improvement with the integration of bio-inspired algorithms:

- The PO algorithm achieves efficiencies of 89.12% (simulation) and 90.54% (real data), highlighting its limitations in adapting to dynamic irradiance changes.
- The PO-GWO algorithm increases efficiency significantly to 97.65% (simulation) and 98.68% (real data), showcasing its capability to track the maximum power point (MPP) with higher accuracy.
- The PO-WOA algorithm achieves the highest efficiency at 98.87% (simulation) and 98.94% (real data), surpassing both PO and PO-GWO. This improvement underscores the superior optimization capabilities of WOA in handling complex and fluctuating conditions.

### Average power loss

Power loss directly reflects the energy not utilized due to suboptimal MPP tracking:

- The PO algorithm records significant power losses of 5.3 W (simulation) and 4.9 W (real data), emphasizing its inefficiency in tracking MPP under variable conditions.
- The PO-GWO algorithm reduces power loss to 1.3 W (simulation) and 1.02 W (real data), benefiting from its adaptive step-size mechanism.
- The PO-WOA algorithm further minimizes power loss to 0.56 W (simulation) and 0.39 W (real data), indicating its ability to maintain optimal energy extraction across diverse scenarios.

### Average response time

Response time is indicative of how quickly an algorithm can adjust to changing irradiance levels:

- The PO algorithm exhibits the shortest response times, at 0.23 s (simulation) and 0.36 s (real data), owing to its fixed-step approach.
- The PO-GWO algorithm has moderate response times of 0.51 s (simulation) and 0.41 s (real data), balancing speed and precision.
- The PO-WOA algorithm displays slightly longer response times of 0.65 s (simulation) and 0.48 s (real data), prioritizing accuracy and stability over rapid adjustments.

**Table 7:** Comparative analysis of MPPT algorithm performance metrics under simulated and real irradiation conditions (June 21, 2023).

	Simulation irradiance profile			Irradiation levels data for June 21, 2023		
	PO	PO-GWO	PO-WOA	PO	PO-GWO	PO-WOA
Avg. Efficiency (%)	89.12	97.65	98.87	90.54	98.68	98.94
Avg. Power loss (W)	5.3	1.3	0.56	4.9	1.02	0.39
Avg. Response time (s)	0.23	0.51	0.65	0.36	0.41	0.48

The data presented in Table 7 highlights the trade-offs among the algorithms. While the PO algorithm provides quick responses, its lower efficiency and higher power loss lim it its suitability for dynamic PV systems. In contrast, PO-GWO and PO-WOA deliver significantly better efficiency and reduced power losses, albeit with slightly slower response times. Among the advanced algorithms, PO-WOA consistently outperforms PO-GWO, making it the preferred choice for applications requiring high precision and energy optimization.

## Conclusion

This study introduces two novel MPPT techniques for PV systems: the GWO algorithm and the WOA, both utilizing adaptive step-size strategies. The investigation includes comprehensive simulations in Simulink, which simulate a variety of environmental conditions. These simulations optimize the gains of the PID controller to enhance the system’s transient response, minimize overshoot, and reduce oscillatory behaviour. The primary contributions of this research are the substantial improvements in the dynamic performance of PV systems, particularly in terms of power tracking efficiency.

Experimental validation, based on real data collected on June 21, 2023, from 08:00 AM to 06:00 PM, further corroborates the effectiveness of the proposed MPPT methods. The fixed-step-size PO controller tracks the maximum power point under varying irradiance conditions but suffers from a significant peak power overshoot of 15.3 W during periods of rapid meteorological changes. In contrast, the adaptive step-size GWO and WOA algorithms exhibit considerably lower power overshoots, 0.9 W and 0.12 W, respectively, under similar conditions. Quantitative analysis of the proposed MPPT algorithms reveals notable performance improvements over traditional fixed-step methods. Specifically, the adaptive step-size algorithms achieve a 90% reduction in ripple, a 60% reduction in overshoot, and a 30% faster response time. These results underscore the efficacy of bio-inspired optimization algorithms in enhancing the precision and reliability of MPPT under dynamically changing environmental conditions, making them highly applicable for real-world PV applications. Table 7 highlights the comparative performance of PO, PO-GWO, and PO-WOA algorithms under both simulated and real irradiation conditions. The findings show that while the PO algorithm exhibits faster response times, its performance is limited by lower efficiency and higher power losses. Conversely, the bio-inspired PO-GWO and PO-WOA algorithms demonstrate superior efficiency and significantly reduced power losses, with PO-WOA consistently outperforming PO-GWO in all metrics. These results underscore the importance of balancing response speed and tracking accuracy for optimizing energy extraction in dynamic PV systems.

Future work will focus on further optimizing the GWO and WOA algorithms to improve their robustness in the face of rapid environmental fluctuations. The integration of machine learning techniques to intelligently adjust the step size and reduce the convergence time will be explored. Real-world validation will be conducted across different PV system configurations and climatic conditions to assess the practical applicability and scalability of the proposed methods. Furthermore, hybrid optimization techniques combining GWO and WOA with other meta-heuristic algorithms will be investigated to further enhance system performance. Expanding these algorithms to other renewable energy systems, such as wind turbines or hybrid PV-wind configurations, will also be a key direction for future research, aiming to contribute to the optimization of sustainable energy solutions.

## Data Availability

The datasets used and/or analysed during the current study available from the corresponding author on reasonable request.
